# Prevalence and factors associated with cryptococcal antigenemia among severely immunosuppressed HIV-infected adults in Uganda: a cross-sectional study

**DOI:** 10.1186/1758-2652-15-15

**Published:** 2012-03-14

**Authors:** Jacinta Oyella, David Meya, Francis Bajunirwe, Moses R Kamya

**Affiliations:** 1 Makerere University College of Health Sciences New Mulago Hospital Complex, Mulago Hill Road, P.O BOX 7072, Kampala, Uganda; 2 Makerere University Infectious Diseases Institute, Kampala, Uganda; 3 Mbarara University of Science and Technology, Mbarara, Uganda; 4 Division of Infectious Disease and International Medicine, Department of Medicine, University of Minnesota, Minneapolis, Minnesota, USA

## Abstract

**Background:**

Cryptococcal infection is a common opportunistic infection among severely immunosuppressed HIV patients and is associated with high mortality. Positive cryptococcal antigenemia is an independent predictor of cryptococcal meningitis and death in patients with severe immunosuppression. We evaluated the prevalence and factors associated with cryptococcal antigenemia among patients with CD4 counts of 100 cells/mm^3 ^or less in Mulago Hospital, Kampala, Uganda. Screening of a targeted group of HIV patients may enable early detection of cryptococcal infection and intervention before initiating antiretroviral therapy. Factors associated with cryptococcal antigenemia may be used subsequently in resource-limited settings in screening for cryptococcal infection, and this data may also inform policy for HIV care.

**Methods:**

In this cross-sectional study, HIV-infected patients aged 18 years and older with CD4 counts of up to 100 cells/mm^3 ^were enrolled between December 2009 and March 2010. Data on socio-demographics, physical examinations and laboratory tests were collected. Factors associated with cryptococcal antigenemia were analyzed using multiple logistic regression.

**Results:**

We enrolled 367 participants and the median CD4 count was 23 (IQR 9-51) cells/mm^3^. Sixty-nine (19%) of the 367 participants had cryptococcal antigenemia. Twenty-four patients (6.5%) had cryptococcal meningitis on cerebrospinal fluid analysis and three had isolated cryptococcal antigenemia. Factors associated with cryptococcal antigenemia included: low body mass index of 15.4 kg/m^2 ^or less (adjusted odds ratio, AOR = 0.5; 95% CI 0.3-1.0), a CD4+ T cell count of less than 50 cells/mm^3 ^(AOR = 2.7; 95% CI1.2-6.1), neck pain (AOR = 2.3; 95% CI 1.2-4.6), recent diagnosis of HIV infection (AOR = 1.9; 95% CI 1.1-3.6), and meningeal signs (AOR = 7.9; 95% CI 2.9-22.1). However, at sub-analysis of asymptomatic patients, absence of neck pain (AOR = 0.5), photophobia (AOR = 0.5) and meningeal signs (AOR = 0.1) were protective against cryptococcal infection.

**Conclusions:**

Cryptococcal antigenemia is common among severely immunosuppressed HIV patients in Mulago Hospital, Kampala, Uganda. Independent predictors of positive serum cryptococcal antigenemia were CD4^+ ^T cell counts of less than 50 cells/mm, low body mass index, neck pain, signs of meningeal irritation, and a recent diagnosis of HIV infection. Routine screening of this category of patients may detect cryptococcosis, and hence provide an opportunity for early intervention. Absence of neck pain, photophobia and meningeal signs were protective against cryptococcal infection compared with symptomatic patients.

## Background

Cryptococcosis is a common and serious infection in patients with advanced human immunodeficiency virus (HIV) infection. Recent estimates suggest that there are about 1 million new cases of cryptococcosis and at least 500,000 deaths annually worldwide due to HIV-associated cryptococcosis [1]. The vast majority of cases occur among patients living in sub-Saharan Africa. The rate of cryptococcal infection in Uganda was 40.4 cases per 1,000 person years and 1.5 cases per 1,000 person years in the pre-highly active antiretroviral therapy (HAART) and HAART period, respectively [2]. In more affluent countries, the incidence of HIV-associated cryptococcosis has decreased dramatically [3], and early mortality associated with this disease is generally under 10%. Among patients who die early upon antiretroviral therapy (ART) initiation, cryptococcal disease is the second most common cause of death after tuberculosis [4]. A cohort in Uganda showed a 14-day mortality rate of 20% to 42% among patients with cryptococcal meningitis (CM) despite treatment with Amphotericin B [5].

In areas of high incidence of cryptococcal disease, primary antifungal prophylaxis is shown to be effective in reducing the incidence of CM [6]. Cost effectiveness of prophylaxis was found at $511 per life year gained[7]. The best prophylaxis is rapid immune reconstitution with ART. However, in areas of high incidence of CM, screening for cryptococcosis prior to ART initiation is necessary for potential early diagnosis and treatment. This could decrease the risk of immune inflammatory syndrome [8,9].

In Cambodia and South Africa, screening for cryptococcal antigenemia in HIV-infected patients with advanced HIV infection predicted mortality and incidence of CM. The prevalence of cryptococcal antigenemia was 18% [10] and 13% [11], respectively. The majority of HIV-infected patients in resource-limited settings present late with advanced immunosuppression.

In this study, we screened HIV-infected patients with CD4 counts of up to 100 cells/mm^3 ^at Mulago Hospital, Kampala, Uganda, to determine the prevalence and factors associated with cryptococcal antigenemia. The serum cryptococcal antigen test has been found to be cost effective in preventing deaths in HIV patients with severe immunosuppression [12]. Screening of a targeted group of HIV patients may enable early detection of cryptococcal infection and intervention before initiating ART. This study will, therefore, inform guidelines for screening, diagnosis and treatment of cryptococcal infection among severely immunosuppressed HIV-infected patients in resource-limited settings.

## Methods

### HIV care and treatment in Mulago Hospital, Uganda

We conducted a cross-sectional study involving both inpatients and outpatients at the 1,500-bed Mulago National Referral and Teaching Hospital, in Kampala, Uganda. Study participants were enrolled from the medical wards and the HIV outpatient clinic between December 2009 and March 2010. Routine counselling and testing for HIV and CD4 count testing are offered in the hospital through PEPFAR support under the Makerere Mbarara Joint AIDS Program on a daily basis in the medical wards. The HIV outpatient clinic is located on the same floor as the medical wards. The clinic provides ART, prophylaxis and management of opportunistic infections, CD4 count testing and other investigations.

### Study design and patients

Patients were only approached to participate in the study if they were HIV positive and had CD4 results that were less than six months old. All HIV-infected adults on the medical wards and patients referred to the outpatient HIV clinic for their first visit were screened and gave informed consent to participate in the study. For very sick patients, informed consent was obtained from the next of kin. Patients were enrolled if they had CD4^+ ^counts of 100 cells/mm^3 ^or less, no prior history of cryptococcosis, were not receiving fluconazole treatment, and were ART naïve. A questionnaire was used to collect data on socio-demographics and medical history. A detailed physical examination was performed. Clinical signs of meningitis were categorized as neck stiffness, altered mental status or neurological deficits.

This study was reviewed and approved by the Makerere School of Medicine Research and Ethics Committee and the Uganda National Council for Science and Technology.

### Definitions

Severe immunosuppression was defined as a CD4+ T cell count of 100 cells/mm^3 ^or less. Cryptococcal antigenemia was defined as having a positive serum *Cryptococcal *antigen test. Cryptococcal meningitis was defined as having one of the following: symptoms and signs of meningitis with a positive CRAG test, positive India ink or serum and cerebrospinal fluid (CSF) culture of *C. neoformans*. Isolated cryptococcal antigenemia was defined by only positive serum cryptococcal antigen by latex agglutination test with negative culture in CSF or negative CSF cryptococcal antigen by latex agglutination test.

*Meningeal signs were *defined as resistance to knee extension with both knee and hip flexed while the patient was supine, and nuchal rigidity.

### Laboratory assays

CSF or serum cryptococcal antigen was detected using a latex agglutination test (Wampole Laboratories Crypto-LA test New Jersey, USA) following the manufacturer's instructions at the Faculty of Medicine Laboratory, Makerere University. Briefly, the specimens' serum or CSF were screened with the detacher enzyme and incubated in tubes at 56°C for 30 minutes. Then, the enzyme inhibitor was added, and the specimen was applied on the agglutination slide and then mixed with anti-cryptococcal latex. The slide was placed on the rotator at 100 revs/minute for five minutes. The reactions were graded as follows: negative result was indicated by a smooth, milky suspension with absence of agglutination; and positive result was indicated as distinct large clumps against a clear or slightly milky background, or small definite clumps against a milky background.

The test kit included a pronase and has been shown to have a sensitivity of 93% to 100% and a specificity of 96% to 98% [13]. CSF specimens for culture of *Cryptococcus neoformans *were centrifuged and the sediment inoculated onto Sabouraud's dextrose agar and incubated at 30°C for 14 days. The suspected colonies were identified by standard methods [14].

### Statistical methods

All analyses were performed using STATA 10.0 (Texas, USA) program. Medians and frequencies (%) were used to describe patients' characteristics. Fisher's exact test was used to compare categorical variables where appropriate. Student's t-test was performed to assess the differences between the two means. Binary logistic regression was used to determine factors associated with positive serum cryptococcal antigen. Factors that were significantly associated with positive serum cryptococcal antigen at bivariate analyses were entered into a multiple logistic regression to determine their independent association. For strength of association, adjusted odds ratios and a p value of ≤ 0.05 were considered significant.

## Results

### Patients' characteristics at admission and initial contact in the clinic

**Figure 1:**
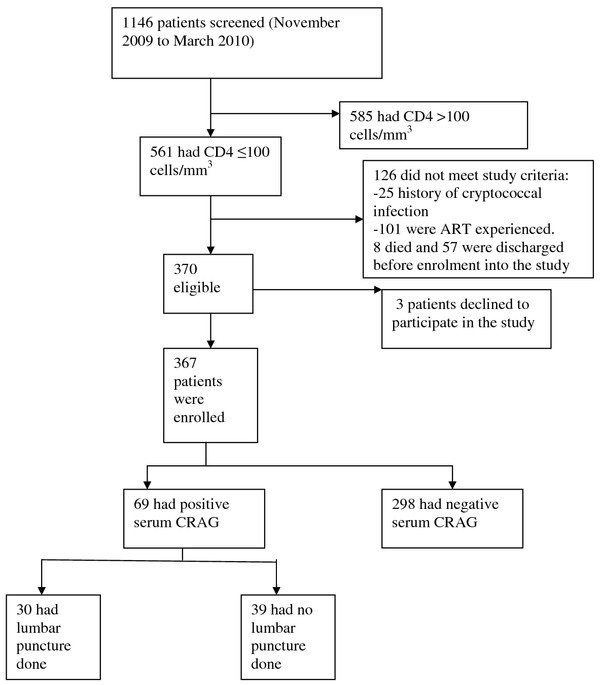
**Study profile**.

**Figure 2:**
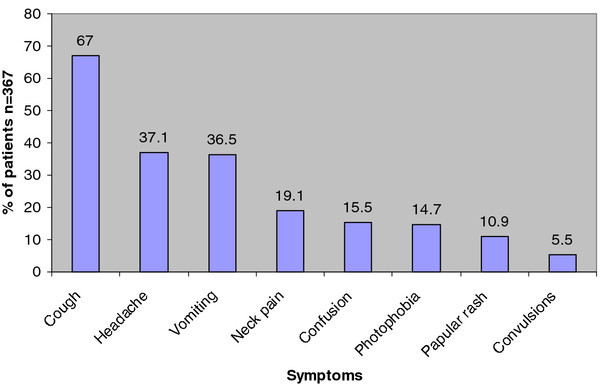
**Common symptoms among study patients (n = 367) screened for serum cryptococcal antigenemia at Mulago Hospital, Kampala**.

Between November 2009 and March 2010, 1,146 HIV-positive patients were screened at Mulago Hospital, Uganda. Only 370 of the patients screened met the eligibility criteria (see Figure 1). In all, 367 patients with a median CD4^+ ^count of 23 (IQR 9-51) cells/mm^3 ^were enrolled (see Table 1). The median age of the patients was 32 (IQR 28-39) years. There were slightly more female (52%) than male (48%) patients. The majority of participants were married (45%), had attained primary education (52%), and lived in urban areas (77%). A total of 97.3% participants were inpatients and 2.7% were outpatients. A median duration in days of presenting symptoms were as follows: cough 30 (IQR 21-60), headache 14 (IQR 7-30), vomiting 7 (IQR 3-21), neck pain 7 (IQR 5-14), confusion 7 (IQR 3-7), photophobia 7 (IQR 5-14), papular rash 90 (IQR 60-180) and convulsion 2 (IQR 2-7) (see Figure 2).

**Table 1:** Baseline characteristics of the patients at enrolment

Characteristics	Frequency (%) N = 367
Age median (IQR) in years	32 (28 to 39)
Sex	
Males	177(48.2)
Females	190(51.8)
Residence	
Urban	283(77.1)
Rural	84(22.9)
BMI kg/m^2^	
> 15.4	295(80.4)
≤ 15.4	72(19.6)
Karnofsky score	
60-100	286(77.9)
≤ 50	81(22.1)
Meningeal signs present	24(6.5)
Presence of focal neurological deficits	10(2.7)
Papular rash	27(7.4)
WHO stage	
I	14(3.8)
II	47(12.8)
III	174(47.4)
IV	132(36.0)
HIV status	
Previously known	236(64.3)
Recent HIV diagnosis	131(35.7)
CD4 (cells/mm^3^)	
Median (IQR) 23 (9 to 51)	
50-100	98(26.7)
< 50	269(73.3)

### Prevalence and factors associated with cryptococcal antigenemia

The prevalence of cryptococcal antigenemia among HIV-infected adults with CD4 counts of up to 100 cells/mm^3 ^was 19% (69 of 367) (95% CI 0.15-0.23) in this study. At bivariate analysis the presence of headache, neck pain, convulsions, photophobia, meningeal irritation signs, WHO stage III and IV, altered mentation and CD4 counts of less than 50 cells/mm^3 ^were significantly associated with cryptococcal antigenemia. Refer to Table 2 for analysis of asymptomatic patients with positive serum cryptococcal antigenemia.

**Table 2:** Bivariate analysis of asymptomatic participants by serum CRAG

Characteristics	Serum CRAG-negative patients (n = 298)	Serum CRAG-positive patients (n = 69)	Crude odds ratio	95% CI	p value
**Residence**	230(77.2)	53(76.8)	1.0	0.6-1.9	0.95
**Urban Rural Symptoms**	68(22.8)	16(23.2)			
Neck pain absent	256(85.9)	41(59.4)	0.2	0.1-0.4	**0.00**
Seizures absent	286(95.9)	61(88.4)	0.3	0.1-0.8	**0.02**
Photophobia absent	267(89.6)	46(66.7)	0.2	0.1-0.4	**0.00**
**Physical signs**					
Meningeal signs absent	291(97.7)	52(75.4)	0.1	0.0-0.2	**0.00**

* Significant at p ≤ 0.05 level

Factors independently associated with positive serum cryptococcal antigenemia included: low body mass index (BMI) of 15.4 kg/m^2 ^or less (adjusted odds ratio, AOR = 0.5; 95% CI 0.3-1.0), a CD4+ T cell count of less than 50 cells/mm^3 ^(AOR = 2.7; 95% CI1.2-6.1), neck pain (AOR = 2.3; 95% CI 1.2-4.6), recent diagnosis of HIV infection (AOR = 1.9; 95% CI 1.1-3.6), and meningeal signs (AOR = 7.9; 95% CI 2.9-22.1).

However, sub-analysis of asymptomatic patients with positive serum cryptococcal antigenemia demonstrated that absence of neck pain (AOR = 0.5), photophobia (AOR = 0.5) and meningeal signs (AOR = 0.1) were protective against cryptococcal infection (see Table 3).

**Table 3:** Multivariate analysis: Factors associated with asymptomatic positive cryptococcal antigenemia

Variables	Unadjusted OR	Adjusted OR	95% CI	p value
Meningeal signs absent	0.1	0.1	0.1-0.4	0.00
Photophobia absent	0.2	0.5	0.2-1.0	0.05
Seizures absent	0.3	0.9	0.3-2.9	0.79
Neck pain absent	0.2	0.5	0.2-1.0	0.05

Further microbiological tests were done in patients with positive serum cryptococcal antigenemia as standard of care. Lumbar punctures were performed to exclude cryptococcal meningitis in patients with positive serum cryptococcal antigen tests. However, only 43.5% of the patients consented to having lumbar punctures. The rest did not have lumbar punctures for various reasons: ran away (one), comatose (one), discharged (18), died (six), and declined (13). India ink, cultures for *Cryptococcus neoformans *and CSF CRAG were performed. Tests done showed 24 patients had cryptococcal meningitis, three had isolated cryptococcal antigenemia, and three could not be classified because of incomplete results. Patients with cryptococcal meningitis were hospitalized for treatment with Amphotericin B. Three patients diagnosed with isolated cryptococcal antigenemia were treated with oral fluconazole (200 mg/day) for 12 weeks. Treatment was given as routine care. No follow up was carried out.

## Discussion

The results from our study demonstrate a high prevalence (19%) of cryptococcal antigenemia in an urban setting among HIV-infected patients with severe immunosuppression. This prevalence is comparable to findings in Cambodia [10]. In a community clinic in a rural setting in Tororo, Uganda, a prevalence of only 5.3% was reported. The low prevalence in this study in eastern Uganda is most likely because only patients who were asymptomatic participated in the study [15]. The high prevalence in our study warrants the need to screen for and diagnose cryptococcal infection among patients with severe immunosuppression prior to initiation of ART. This may prevent unmasking of sub-clinical infection, especially in patients who are asymptomatic [16].

Screening for serum cryptococcal antigen is highly sensitive, specific and cost effective in preventing death in HIV-infected patients with CD4 counts of 100 cells/mm^3 ^or less [12]. Cost effectiveness analysis in Uganda suggests that the number needed to test and treat with CRAG screening and fluconazole treatment to prevent one CM case is 11.3 (95% CI 7.9-17.1) at a cost of US$190 (95% CI, $132-$287) while the number needed to test and treat to save one life is 15.9 (95% CI 11.1-24.0) at a cost of $266 (95% CI $185-$402) [12].

Several cohorts in sub-Saharan Africa have reported high early mortality and immune reconstitution inflammatory syndrome after ART initiation [4,16]. In one cohort, a high early mortality rate of 14% during the first year of therapy, particularly during the first three months, was reported [4]. Tuberculosis and cryptococcal disease accounted for at least one-third of all HIV-related deaths. Cryptococcal antigen test is available in a few centres in Uganda and is also very expensive. If this test is made widely available and rolled out as part of HIV care policy, batch testing of samples could be done with a reduction in costs, and thus making it affordable to ensure timely initiation of appropriate management for cryptococcal infection.

The following factors were independently associated with cryptococcal antigenemia: severe immunosuppression with CD4 counts of less than 50 cells/mm^3^, low BMI with less than 15.4 kg/m^2^, meningeal signs, neck pain, and having a recent diagnosis of HIV infection. And asymptomatic patients are more protected against cryptococcal infection, as seen in the analysis. This is the category of patients who may benefit from primary prophylaxis.

Patients with CD4 counts of less than 50 cells/mm^3 ^were more likely to have cryptococcal antigenemia (23% compared with 8% of patients with CD4 counts of more than 50 cells/mm^3^). This finding is similar to that reported by Micol *et al *[10]. Low CD4^+ ^cell counts predispose these patients to cryptococcal infection because of dysfunctional immune systems. In a resource-limited setting, screening of patients with CD4^+ ^counts of less than 50 cells/mm^3 ^for cryptococcal infection may even be more clinically relevant as CD4 tests become more available.

Primary fluconazole prophylaxis among HIV-infected patients is known to be beneficial in preventing cryptococcal disease; however, it is more logical and cost effective to offer targeted intervention with fluconazole for severely immunosuppressed and asymptomatic patients testing positive for cryptococcal antigen [6]. This study has demonstrated that asymptomatic patients are protected against cryptococcal infection compared with symptomatic patients.

In this study, low BMI was independently associated with positive serum CRAG. Malnutrition predisposes to a dysfunctional immune system [17], which could have also predisposed patients to the risk of cryptococcal infection. The following findings have been reported in malnourished HIV-infected patients: suppression of antigen specific arms of immune system and several generalized host defense mechanisms, including decreased T cell primary antibody response, memory response and atrophy of lymph tissues [18]. Peripheral lymphocytes and natural killer cells activities have also been found to be reduced in these patients. Low BMI (under 15.4 kg/m^2^) was reported to be an independent factor associated with cryptococcal antigenemia in Cambodia [10].

One-quarter of the patients with cryptococcal antigenemia had signs of meningeal irritation in our study. The majority of patients with meningeal signs (14 of 17) had CM. Although serum CRAG identifies cryptococcal infection, presence of meningeal signs and neck pain indicates CM. French *et al *noted that cryptococcal antigenemia preceded symptoms of CM by a median duration of 22 days [2]. Neck pain was also found to be independently associated with cryptococcal antigenemia in our study. Neck pain is probably a continuum of meningeal irritation in our patients. The majority of patients with neck pain (22 of 28 or 78.6%) had CM. Severe immunosuppression predisposed these patients to developing stage IV AIDS events.

Nearly half of the patients with cryptococcal antigenemia were newly diagnosed with HIV infection. Patients in our setting still continue to present for the first time with severe immunosuppression and life-threatening events. In a South African cohort, 25 patients (54%) presented with incident cryptococcal antigenemia prior to ART initiation [11]. The need to increase routine counselling and testing in our setting cannot be overemphasized as it offers the opportunity for early screening of cryptococcal infection and other opportunistic infections and thus targeted interventions that in the long run offset the high costs of managing full-blown infections and possibly death.

Headache was found to be insignificant in this study. Culturally, in our setting, headache may be perceived as a state of being unwell. Other co-morbidities could have also presented as headache.

## Conclusions

Our study has shown that almost 20% of severely immunosuppressed HIV-infected patients have positive serum cryptococcal antigenemia. Clinicians should be more vigilant in screening for cryptococcal disease among HIV-infected patients with low CD4 counts (less than 50 cells/mm^3^), presence of meningeal irritation, neck pain, a low BMI (less than 15.4 kg/m^2^), and a recent HIV diagnosis before ART initiation for appropriate intervention. And asymptomatic patients are more protected against cryptococcal infection than symptomatic patients. This is the category of persons who may benefit from primary antifungal prophylaxis.

## Competing interests

The authors declare that they have no competing interests.

## Authors' contributions

JO contributed to conception and design of the study. JO also carried out acquisition, analysis and interpretation of data. She participated in drafting the manuscript and revising it. DM contributed to conception and design of the study. DM also participated in data analysis and interpretation. DM critically revised the manuscript. FB participated in study design, statistical analysis and interpretation of data. FB critically revised the manuscript. MRK contributed to conception and design of the study with crucial critics. MRK carried out data analysis and interpretation. MRK critically revised the manuscript. All authors read and approved the final version of this manuscript.
